# On the convergence of a high-accuracy compact conservative scheme for the modified regularized long-wave equation

**DOI:** 10.1186/s40064-016-2085-9

**Published:** 2016-04-18

**Authors:** Xintian Pan, Luming Zhang

**Affiliations:** School of Mathematics and Information Science, Weifang University, Weifang, 261061 China; School of Management, Qufu Normal University, Rizhao, 276800 China; Department of Mathematics, Nanjing University of Aeronautics and Astronautics, Nanjing, 210016 China

**Keywords:** The MRLW equation, Compact conservative method, Solvability, Convergence, Stability

## Abstract

In this article, we develop a high-order efficient numerical scheme to solve the initial-boundary problem of the MRLW equation. The method is based on a combination between the requirement to have a discrete counterpart of the conservation of the physical “energy” of the system and finite difference method. The scheme consists of a fourth-order compact finite difference approximation in space and a version of the leap-frog scheme in time. The unique solvability of numerical solutions is shown. A priori estimate and fourth-order convergence of the finite difference approximate solution are discussed by using discrete energy method and some techniques of matrix theory. Numerical results are given to show the validity and the accuracy of the proposed method.

## Background

The generalized regularized long-wave (GRLW) equation reads (Peregrine 1996):

1 $$ u_t-\mu u_{xxt}+ u_x+\alpha u^pu_x=0,$$

where $\alpha$ and $\mu$ are positive constants, $p\ge 1$ is an integer. When $p=1$, the Eq. (1) is usually called as the regularized long-wave (RLW) equation proposed by Peregrine (1996) and Benjamin et al. (1972) to describe nonlinear dispersive waves. Various numerical techniques have been developed to solve this equation. These partly include finite difference method, finite element methods, least squares method and collocation method with quadratic B-splines, cubic B-splines and septic splines, multisymplectic numerical method, in this respect, we refer readers to Kutluay and Esen (2006), Zhang (2005), Avilez-Valente and Seabra-Santos (2004), Esen and Kutluay (2006), Guo and Chen (2006); Gu and Chen 2008), Saka and Dag (2008), Dag (2000), Dag and Ozer (2001), Dag et al. (2004), Soliman and Raslan (2001), Soliman and Hussien (2005), Cai (2009, 2011), and references therein. Another special case of Eq. (1) for $p=2$, the modified regularized long-wave (MRLW) equation is given by

2 $$ u_t-\mu u_{xxt}+ u_x+\alpha u^2u_x=0.$$

In recent years, the MRLW equation has attracted much attention of many researchers. Many mathematical and numerical studies have been developed for the MRLW equation in the literatures. Along the mathematical front, for the exact solutions via double reduction theory and Lie symmetries, the bifurcation and travelling wave solutions as well as some explicit analytic solutions obtained from dynamical systems theory, numerical solutions with high degree of accuracy by the variational iteration method and the Adomian decomposition method, we refer readers to Naz et al. (2013), Yan et al. (2012), Labidi and Omrani (2011), Khalifa et al. (2008a).

Along the numerical front, many efficient numerical methods have been developed for the MRLW equation, such as a new ten-point multisymplectic explicit numerical method (Cai 2010), Sinc-collocation method (Mokhtari and Mohammadi 2010), split least-squares mixed finite element method (Gao et al. 2015), B-spline finite element method (Gardner et al. 1997), finite difference method (Khalifa et al. 2007; Akbari and Mokhtari 2014), cubic B-spline collocation method (Khalifa et al. 2008b), quadratic B-spline collocation method (Tirmizi 2010), quadratic B-spline collocation method (Raslan 2009).

In recent works (Dehghan et al. 2009; Xie et al. 2009; Wang and Guo 2011; Wang 2014, 2015), the fourth-order compact finite difference approximation solutions to solve the Klein–Gordon equation, the Schrödinger equation and Klein–Gordon–Schrödinger equation were shown, respectively. The numerical results are encouraging. Motivated by the techniques of these works, in this paper, we propose a linearized compact conservative difference scheme with high accuracy to solve the MRLW equation (2) numerically. The presented compact difference scheme is three-level, linear-implicit and second-order accuracy in time and fourth-order accuracy in space. By means of the matrix theory, we convert the proposed scheme into the vector difference one. The coefficient matrices of the present scheme are symmetric and tridiagonal, and Thomas algorithm can be employed to solve them effectively. Numerical example on the model problem shows that the present scheme is of high accuracy and good stability, which preserves the original conservative properties at the same time.

The rest of this paper is organized as follows. In “The high-accuracy compact conservative vector difference scheme” section, a linearized compact finite difference scheme for the MRLW equation is described. In “Discrete conservative property, estimate and solvability” section, we discuss the solvability of the scheme and the estimate of the difference solution. In “Convergence and stability of the difference scheme” section, convergence and stability of the scheme are proved by using energy method. In “Numerical experiments” section, numerical experiments are reported to test the theoretical results.

## The high-accuracy compact conservative vector difference scheme

In this section, we describe a high-order linear-compact conservative difference scheme for the Eq. (2). Consider the MRLW equation

3 $$ u_t-\mu u_{xxt}+ u_x+\alpha u^2u_x=0,$$

with an initial condition

4 $$ u(x,0)=u_0(x),\quad x\in [x_l,x_r],$$

and the boundary conditions

5 $$ u(x_l,t)=u(x_r,t)=0,\quad t\in [0,T]. $$

where $u_0(x)$ is a known smooth function.

The IBV problem (3)–(5) is known to possess the following conservative property:

6 $$ E(t)=||u||^2_{L_2}+\mu ||u_{x}||^2_{L_2}=E(0). $$

Let $h=\frac{x_r-x_l}{J}$ and $\tau =\frac{T}{N}$ be the uniform step size in the spatial and temporal direction, respectively. Denote $x_j=jh\,(0\le j\le J), t_n=n\tau \,(0\le n\le N)$, $u^n_j\approx u(x_j,t_n)$ and $Z^0_h=\{u=(u_j)|u_0=u_J=0, j=0,1,2,\ldots , J\}$. Define

$$\begin{aligned}&\delta _xu^n_j=\dfrac{u^n_{j+1}-u^n_j}{h},\quad \delta _{\bar{x}}u^n_j=\dfrac{u^n_{j}-u^n_{j-1}}{h},\quad \delta _{\hat{x}}u^n_j=\dfrac{u^n_{j+1}-u^n_{j-1}}{2h},\quad \delta _tu^n_j=\dfrac{u^{n+1}_{j}-u^n_j}{\tau },\\&\delta _{\hat{t}}u^n_j=\dfrac{u^{n+1}_{j}-u^{n-1}_{j}}{2\tau },\quad \bar{u}^n_j=\dfrac{u^{n+1}_j+u^{n-1}_j}{2},\\ &{\mathcal{A}}_hu^n_j=u^n_j+\frac{h^2}{12}\delta _x\delta _{\bar{x}}u^n_j=\frac{1}{12}\left(u^{n}_{j-1}+10u^{n}_j+u^{n}_{j+1}\right)  \\&u^{n+\frac{1}{2}}_j=\dfrac{u^{n+1}_j+u^{n}_j}{2},\quad {\mathcal{B}}_hu^n_j=u^n_j+\frac{h^2}{6}\delta _x\delta _{\bar{x}}u^n_j=\frac{1}{6}\left(u^{n}_{j-1}+4u^{n}_j+u^{n}_{j+1}\right). \end{aligned}$$

In the paper, *C* denotes a general positive constant which may have different values in different occurrences.

For the one-order derivative $u_{x}$ and two-order derivative $u_{xx}$, we have the following formulas:

$$ u_{x}(x_j)={\mathcal{B}}_h^{-1}\delta _{\hat{x}}u(x_j)+O\left( h^4\right) ,\quad N_{xx}(x_j)={\mathcal{A}}_h^{-1}\delta _x\delta _{\bar{x}}u(x_j)+O\left( h^4\right) ,\quad (j\ne 0,J). $$

Omitting the high-order terms $O(h^4)$ in the formulas above, we consider the following three-level linear compact scheme for the IBV problem (3)–(5).

7 $$\begin{aligned}&{\mathcal{A}}_h{\mathcal{B}}_h\delta _{\hat{t}}u^n_j-\mu {\mathcal{B}}_h\delta _x\delta _{\bar{x}}\delta _{\hat{t}}u^n_j+{\mathcal{A}}_h\delta _{\hat{x}}\left( u^n_j\right) +\dfrac{1}{4}\alpha {\mathcal{A}}_h\left\{ \left( \delta _{\hat{x}}\bar{u}^n_j\right) \left( u^n_j\right) ^2+\delta _{\hat{x}}\left[ \left( u^n_j\right) ^2\left( {\bar{u}}^n_j\right) \right] \right\} =0,\nonumber \\&\quad \qquad 1\le j\le J-1, 1\le n\le N-1, \end{aligned}$$

8 $$\begin{aligned}&{\mathcal{A}}_h{\mathcal{B}}_h\delta _tu^0_j-\mu {\mathcal{B}}_h\delta _x\delta _{\bar{x}}\delta _tu^0_j+{\mathcal{A}}_h\delta _{\hat{x}}u^{0+\frac{1}{2}}_j +\alpha \dfrac{1}{4}{\mathcal{A}}_h \left\{ \left( \delta _{\hat{x}}u^{0+\frac{1}{2}}_j\right) \left( u^{0+\frac{1}{2}}_j\right) ^2+\delta _{\hat{x}} \left[ \left( u^{0+\frac{1}{2}}_j\right) ^3\right] \right\} =0,\nonumber \\&\quad \qquad 1\le j\le J-1, \end{aligned}$$

9 $$u^0_j=u_0(x_j),\quad 1\le j\le J, $$

10 $$u^n_0=u^n_J=0,\quad 0\le n\le N. $$

The scheme (7) is three-level and linear-implicit, so it can be easily implemented and suitable for parallel computing.

Define

$$\begin{aligned} {\mathbf{u}}^n&=  {} \left(u^n_{1},u^n_{2},\ldots ,u^n_{J-1}\right)^{\mathrm{T}},\quad {{\varvec{M}}}=\left( {\begin{array}{*{10}{c}} \frac{10}{12} &{}\quad \frac{1}{12} &{}\quad 0 &{}\quad \cdots &{}\quad 0 \\ \frac{1}{12} &{}\quad \frac{10}{12} &{}\quad \frac{1}{12} &{}\quad \cdots &{}\quad 0 \\ &{}\quad \ddots &{}\quad \ddots &{}\quad \ddots \\ 0 &{}\quad \cdots &{}\quad \frac{1}{12} &{}\quad \frac{10}{12} &{}\quad \frac{1}{12} \\ 0 &{}\quad \cdots &{}\quad 0 &{}\quad \frac{1}{12} &{}\quad \frac{10}{12} \\ \end{array}} \right) _{(J-1)\times (J-1)};\\ {\varvec{K}}&=  {} \left( {\begin{array}{*{10}{c}} \frac{4 }{6} &{}\quad \frac{1}{6} &{}\quad 0 &{}\quad \cdots &{}\quad 0 \\ \frac{1}{6} &{}\quad \frac{4 }{6} &{}\quad \frac{1}{6} &{}\quad \cdots &{}\quad 0 \\ &{}\quad \ddots &{}\quad \ddots &{}\quad \ddots \\ 0&{}\quad \cdots &{}\quad \frac{1}{6} &{}\quad \frac{4 }{6} &{}\quad \frac{1}{6} \\ 0&{}\quad \cdots &{}\quad 0 &{}\quad \frac{1 }{6} &{}\quad \frac{4}{6} \\ \end{array}} \right) _{(J-1)\times (J-1)}. \end{aligned}$$

Notice that ${\varvec{M}}$ and ${\varvec{K}}$ are two real-value symmetric positive definite matrices. Hence there exist two real-value symmetric positive definite matrices ${\varvec{G}}$ and ${\varvec{H}}$, such that ${\varvec{G}}= {\varvec{M}}^{-1}$, ${\varvec{H}}= {\varvec{K}}^{-1}$. Then (7)–(10) can be rewritten into the vector form as follows:

11 $$\begin{aligned}&\delta _{\hat{t}}{\mathbf{u}}^n-\mu {{\varvec{G}}}\delta _x\delta _{\bar{x}}\delta _{\hat{t}}{\mathbf{u}}^n+{\varvec{H}}\delta _{\hat{x}}{\mathbf{u}}^n +\dfrac{1}{4}\alpha {\varvec{H}}\left\{ \left( \delta _{\hat{x}}\bar{{\mathbf{u}}}^n\right) \left({\mathbf{u}} ^n\right) ^2+\delta _{\hat{x}}\left[ \left( {\mathbf{u}} ^n\right) ^2\bar{{\mathbf{u}}}^n\right] \right\} =0,\\ &\quad 1\le n\le N-1, \end{aligned}$$

12 $$\begin{aligned}&\delta _t{\mathbf{u}}^0-\mu {{\varvec{G}}}\delta _x\delta _{\bar{x}}\delta _t{\mathbf{u}}^0+{{\varvec{H}}}\delta _{\hat{x}}{\mathbf{u}}^{0+\frac{1}{2}} +\dfrac{1}{4}\alpha {\varvec{H}}\left\{ \left( \delta _{\hat{x}}{\mathbf{u}}^{0+\frac{1}{2}}\right) \left({\mathbf{u}}^{0+\frac{1}{2}}\right) ^2\right.\\ &\left.\quad +\delta _{\hat{x}}\left[\left({\mathbf{u}} ^{0+\frac{1}{2}}\right) ^3\right] \right\} =0, \end{aligned}$$

13 $${\mathbf{u}}^0={\mathbf{u}}_0,\quad 1\le j\le J-1, $$

14 $$\begin{aligned}&u^n_0=u^n_J=0,\quad 0\le n\le N. \end{aligned}$$

For convenience, the last term of (11) is defined by

$$\begin{aligned} \kappa \left( {\mathbf{u}}^n,{\bar{{\mathbf{u}}}}^n\right) =\dfrac{1}{4}\alpha {\varvec{H}}\left\{ \left( \delta _{\hat{x}}\bar{{\mathbf{u}}}^n\right) \left( {\mathbf{u}}^n\right) ^2+\delta _{\hat{x}}\left[ \left( {\mathbf{u}}^n\right) ^2\bar{{\mathbf{u}}}^n\right] \right\} . \end{aligned}$$

## Discrete conservative property, estimate and solvability

In this section, we shall discuss the estimate for the difference solution and the solvability of the difference scheme (11). For $\forall v^n, w^n\in Z^0_h$, we define the discrete inner products and norms on $Z^0_h$ via:

$$\begin{aligned}&\left( v^n,w^n\right) =h\sum _{j=1}^{J-1}v^n_j{\overline{w^n_j}},\quad \left( \delta _x v^n,\delta _x w^n\right) _l=h\sum _{j=0}^{J-1}\delta _xv^n_j\delta _x{\overline{w^n_j}},\quad ||v^n||^2=\left( v^n,v^n\right) ,\\&||\delta _xv^n||=\sqrt{\left( \delta _xv^n,\delta _xv^n\right) _l},\quad ||v^n||_\infty =\max _{1\le j\le J-1}|v^n_j|. \end{aligned}$$

To analyze the discrete conservative property and estimates of difference solution for the scheme (11)–(14), the following lemmas should be introduced.

### **Lemma 1**

(Wang and Guo 2011) *For any real value symmetric positive definite matrix*${{\varvec{G}}}_{(J-1)\times (J-1)}$*, then we have*

$$\begin{aligned}&\left( {\varvec{G}}\delta _x\delta _{\bar{x}}{} {\mathbf{u}} ^n,{\mathbf{u}} ^n\right) =-||{\varvec{R}}\delta _x{\mathbf{u}} ^n||^2,\\&\left( {\varvec{G}}\delta _x\delta _{\bar{x}}\left( {\mathbf{u}} ^{n+1}+{\mathbf{u}} ^{n-1}\right) ,{\mathbf{u}} ^{n+1}-{\mathbf{u}} ^{n-1}\right) =-\left( ||{\varvec{R}}\delta _x{\mathbf{u}} ^{n+1}||^2-||{\varvec{R}}\delta _x{\mathbf{u}} ^{n-1}||^2\right) , \end{aligned}$$

*where*${\varvec{R}}$*is obtained from*${\varvec{G}}$*by Cholesky decomposition* (Zhang 2004).

### **Lemma 2**

(Wang 2015) *On the matrices*${\varvec{M}}$*and*${\varvec{K}}$*, there are the following results:*

1. *The eigenvalues of the matrices*${\varvec{M}}$*and*${\varvec{K}}$*are*
  $$ \lambda _{M,j}=\frac{1}{12}\left( 10+2\cos \frac{j\pi }{J}\right) ,\quad \lambda _{K,j}=\frac{1}{6}\left( 4+2\cos \frac{j\pi }{J}\right) ,\quad j=1,2,\ldots ,J-1. $$
2. *The two matrices have the same eigenvectors*
  $$\begin{aligned} \mathbf{v }_k= \left( \sin \frac{k\pi }{J},\sin \frac{2k\pi }{J},\ldots ,\sin \frac{(J-1)k\pi }{J}\right) ^{\mathrm{T}}, \quad k=1,2,\ldots ,J-1. \end{aligned}$$

### **Lemma 3**

*For real value symmetric positive definite matrices*${\varvec{G}}_{(J-1)\times (J-1)}={\varvec{M}}^{-1}$*and*${\varvec{H}}_{(J-1)\times (J-1)}={\varvec{K}}^{-1}$*, then there exist three positive constants*$C_0$*,*$C_1$*and*$C_2$*, such that*

15 $$C_0||{\mathbf{u}} ^n||^2\le \left( {\varvec{G}}{} {\mathbf{u}} ^n,{\mathbf{u}} ^n\right) =||{\varvec{R}}{} {\mathbf{u}} ^n||^2\le C_1||{\mathbf{u}} ^n||^2,$$

16 $$C_0||{\mathbf{u}} ^n||^2\le \left( {\varvec{H}}{} {\mathbf{u}} ^n,{\mathbf{u}} ^n\right) =||{\varvec{S}}{} {\mathbf{u}} ^n||^2\le C_2||{\mathbf{u}} ^n||^2, $$

*where*$$C_0=1,C_1=\frac{3}{2},C_2=3,$$$${\varvec{R}}$$*and*$${\varvec{S}}$$*are obtained from*$${\varvec{G}},$$$${\varvec{H}}$$*by Cholesky decomposition* (Zhang 2004) *respectively*.

### *Proof*

It follows from Lemma 2 that

17 $$ \frac{2}{3}\le |\lambda _{M,j}|\le 1,\quad \frac{1}{3}\le |\lambda _{K,j}|\le 1. $$

This implies that

18 $$ 1\le |\lambda _{G,j}|\le \frac{3}{2},\quad  1\le |\lambda _{H,j}|\le 3.$$

Notice that ${\varvec{G}}$ and ${\varvec{H}}$ are also real value symmetric positive definite matrices. From Cholesky decomposition, we obtain

19 $$ {\varvec{G}}={\varvec{R}}^{\mathrm{T}}{\varvec{R}}, {\varvec{H}}={\varvec{S}}^{\mathrm{T}}{\varvec{S}}.$$

Then

20 $$ \left( {\varvec{G}}{} {\mathbf{u}} ^n,{\mathbf{u}} ^n\right) =\left( {\varvec{R}}{} {\mathbf{u}} ^n,{\varvec{R}}{} {\mathbf{u}} ^n\right) =||{\varvec{R}}{} {\mathbf{u}} ^n||^2. $$

This together with the definition of matrix norm and (18) gives that

21 $$\begin{aligned} C_0||{\mathbf{u}} ^n||^2\le ||{\varvec{R}}{} {\mathbf{u}} ^n||^2\le C_1||{\mathbf{u}} ^n||^2, \end{aligned}$$

where $C_0=1,C_1=\frac{3}{2}$. Similarly, we can also obtain

22 $$ C_0||{\mathbf{u}} ^n||^2\le ||{\varvec{S}}{} {\mathbf{u}} ^n||^2\le C_2||{\mathbf{u}} ^n||^2, $$

where $$C_0=1,C_2=3$$.

### *Remark 1*

On the above real value symmetric positive definite matrices ${\varvec{G}}$ and ${\varvec{H}}$, according to Lemmas 2 and 3, for *C* is big enough, we can have $||{\varvec{S}}{} {\mathbf{u}} ^n||^2\le C||{\varvec{R}}{} {\mathbf{u}} ^n||^2$.

We also use the following Lemma.

### **Lemma 4**

(Discrete Sobolev’s inequality Zhou 1990) *There exist two positive constants*$C_1$*and*$C_2$*such that*

$$\begin{aligned} ||u^n||_{\infty }\le C_1||u^n||+C_2||\delta _xu^n||. \end{aligned}$$

### **Theorem 1**

*Suppose*$u_0\in H^1_0[x_l,x_r]$*, then the scheme *(11)–(14) *admits the following invariant*

23 $$\begin{aligned} E^n&=\frac{1}{2}\left( ||{\mathbf{u}} ^{n+1}||^2+||{\mathbf{u}} ^{n}||^2\right) +\frac{1}{2}\mu \left(||{\varvec{R}}\delta _x{\mathbf{u}} ^{n+1}||^2+||{\varvec{R}}\delta _x{\mathbf{u}} ^n||^2\right)\\& \quad+ h\tau \sum _{j=1}^{J-1}\left({\varvec{S}}\delta _{\hat{x}}u_j^{n}\right){\varvec{S}}u^{n+1}_j\\ &=E^{n-1}=\cdots =E^0.\end{aligned}$$

### *Proof*

Taking the inner product of (11) with ${\mathbf{u}} ^{n+1}+{\mathbf{u}} ^{n-1}$ (i.e. $2\bar{{\mathbf{u}}}^n$) and using Lemma 1 yield

24 $$\begin{aligned}&\frac{1}{2\tau }\left( ||{\mathbf{u}} ^{n+1}||^2-||{\mathbf{u}} ^{n-1}||^2\right) +\frac{1}{2\tau }\mu \left( ||{\varvec{R}}\delta _x{\mathbf{u}} ^{n+1}||^2-||{\varvec{R}}\delta _x{\mathbf{u}} ^{n-1}||^2\right) +\left( {\varvec{H}}\delta _{\hat{x}}{} {\mathbf{u}} ^{n},{\mathbf{u}} ^{n+1}+{\mathbf{u}} ^{n-1}\right) \nonumber \\&\quad +\,\left( \kappa \left( {\mathbf{u}} ^n,\bar{{\mathbf{u}}}^n\right) ,2\bar{{\mathbf{u}}}^n\right) =0. \end{aligned}$$

Computing the third term of the left-hand side in (24), we get

25 $$\begin{aligned} \left( {\varvec{H}}\delta _{\hat{x}}{} {\mathbf{u}} ^{n},{\mathbf{u}} ^{n+1}+{\mathbf{u}} ^{n-1}\right)&=\left[ \left( {\varvec{S}}\delta _{\hat{x}}{} {\mathbf{u}} ^{n},{\varvec{S}}{} {\mathbf{u}} ^{n+1}\right) -\left( {\varvec{S}}{} {\mathbf{u}} ^{n},{\varvec{S}}\delta _{\hat{x}}{} {\mathbf{u}} ^{n-1}\right) \right] \nonumber \\&=\left[ h\sum_{j=1}^{J-1}\left( {\varvec{S}}\delta _{\hat{x}}u_j^{n}\right) {\varvec{S}}u^{n+1}_j-h\sum _{j=1}^{J-1}\left( {\varvec{S}}\delta _{\hat{x}}u_j^{n-1}\right) {\varvec{S}}u^{n}_j\right] . \end{aligned}$$

Computing the fourth term of the left-hand side in (24) yields

26 $$\begin{aligned} \left( \kappa \left( {\mathbf{u}} ^n,\bar{{\mathbf{u}}}^n\right) ,2\bar{{\mathbf{u}}}^n\right)&=\left( \dfrac{1}{4}\alpha {\varvec{H}}\left\{ \left( \delta _{\hat{x}}\bar{{\mathbf{u}}}^n\right) \left( {\mathbf{u}} ^n\right) ^2+\delta _{\hat{x}}\left[ \left( {\mathbf{u}} ^n\right) ^2{\bar{{\mathbf{u}}}}^n\right] \right\} ,2\bar{{\mathbf{u}}}^n\right) \nonumber \\&=\dfrac{1}{2}\alpha \left[ \left( {\varvec{S}}\left( \delta _{\hat{x}}\bar{{\mathbf{u}}}^n\right) \left( {\mathbf{u}} ^n\right) ^2,{\varvec{S}}\bar{{\mathbf{u}}}^n\right) -\left( {\varvec{S}}\left( {\mathbf{u}} ^n\right) ^2\bar{{\mathbf{u}}}^n,{\varvec{S}}\delta _{\hat{x}}\bar{{\mathbf{u}}}^n\right) \right] \nonumber \\&=\dfrac{1}{2}\alpha \left[ \left( {\varvec{H}}\left( \delta _{\hat{x}}\bar{{\mathbf{u}}}^n\right) \left( {\mathbf{u}} ^n\right) ^2,\bar{{\mathbf{u}}}^n\right) -\left( {\varvec{S}}\left( {\mathbf{u}} ^n\right) ^2\bar{{\mathbf{u}}}^n,{\varvec{S}}\delta _{\hat{x}}\bar{{\mathbf{u}}}^n\right) \right] \nonumber \\&=\dfrac{1}{2}\alpha \left[ h\sum _{j=1}^{J-1}{\varvec{H}}\left( \delta _{\hat{x}}\bar{u}^n_j\right) \left( u^n_j\right) ^2\bar{u}^n_j-\left( {\varvec{S}}\left( {\mathbf{u}} ^n\right) ^2\bar{{\mathbf{u}}}^n,{\varvec{S}}\delta _{\hat{x}}\bar{{\mathbf{u}}}^n\right) \right] \nonumber \\&=\dfrac{1}{2}\alpha \left[ {\varvec{H}}\left( \delta _{\hat{x}}\bar{{\mathbf{u}}}^n,\left( {\mathbf{u}} ^n\right) ^2\bar{{\mathbf{u}}}^n\right) -\left( {\varvec{S}}\left( {\mathbf{u}} ^n\right) ^2\bar{{\mathbf{u}}}^n,{\varvec{S}}\delta _{\hat{x}}\bar{{\mathbf{u}}}^n\right) \right] \nonumber \\&=\dfrac{1}{2}\alpha \left[ \left( {\varvec{S}}\delta _{\hat{x}}\bar{{\mathbf{u}}}^n,{\varvec{S}}\left( {\mathbf{u}} ^n\right) ^2\bar{{\mathbf{u}}}^n\right) -\left( {\varvec{S}}\left( {\mathbf{u}} ^n\right) ^2\bar{{\mathbf{u}}}^n,{\varvec{S}}\delta _{\hat{x}}\bar{{\mathbf{u}}}^n\right) \right] =0. \end{aligned}$$

It follows from (24) to (26) that

27 $$\begin{aligned}&\frac{1}{2\tau }\left( ||{\mathbf{u}} ^{n+1}||^2-||{\mathbf{u}} ^{n-1}||^2\right) +\frac{1}{2\tau }\mu \left( ||{\varvec{R}}\delta _x{\mathbf{u}} ^{n+1}||^2-||{\varvec{R}}\delta _x{\mathbf{u}} ^{n-1}||^2\right)\\ &\quad + h\sum _{j=1}^{J-1}\left( {\varvec{S}}\delta _{\hat{x}}u_j^{n}\right) {\varvec{S}}u^{n+1}_j \nonumber  - h\sum _{j=1}^{J-1}\left( {\varvec{S}}\delta _{\hat{x}}u_j^{n-1}\right) {\varvec{S}}u^{n}_j= 0. \end{aligned}$$

Thus

28 $$\begin{aligned} E^n= E^{n-1}=\cdots =E^0, \end{aligned}$$

where $E^n=\frac{1}{2}(||{\mathbf{u}} ^{n+1}||^2+||{\mathbf{u}} ^{n}||^2)+\frac{1}{2}\mu (||{\varvec{R}}\delta _x{\mathbf{u}} ^{n+1}||^2+||{\varvec{R}}\delta _x{\mathbf{u}} ^n||^2)+ h\tau \sum \nolimits _{j=1}^{J-1}({\varvec{S}}\delta _{\hat{x}}u_j^{n}){\varvec{S}}u^{n+1}_j$. This completes the proof of Theorem 1.

### **Theorem 2**

*Assume that*$u_0$*is sufficiently smooth, then there is the estimation for the solution*${\mathbf{u}} ^n$*of the scheme* (11)–(14): $||{\mathbf{u}} ^n||\le C, ||\delta _x{\mathbf{u}} ^n||\le C$, which yield $||{\mathbf{u}} ^n||_{\infty }\le C$.

### *Proof*

It follows from (23) that

29 $$ \frac{1}{2}\left( ||{\mathbf{u}} ^{n+1}||^2+||{\mathbf{u}} ^{n}||^2\right) +\frac{1}{2}\mu \left( ||{\varvec{R}}\delta _x{\mathbf{u}} ^{n+1}||^2+||{\varvec{R}}\delta _x{\mathbf{u}} ^n||^2\right) \le C+\frac{\tau }{2}\left( ||{\varvec{S}}\delta _x{\mathbf{u}} ^n||^2+||{\varvec{S}}{} {\mathbf{u}} ^{n+1}||^2\right) .$$

This together with Lemma 3 gives that

30 $$ \frac{1}{2}\left[ (1-C_2\tau )||{\mathbf{u}} ^{n+1}||^2+||{\mathbf{u}} ^{n}||^2\right] +\frac{1}{2}\left[ \mu ||{\varvec{R}}\delta _x{\mathbf{u}} ^{n+1}||^2+\left( C_0\mu -C_2\tau \right) ||\delta _x{\mathbf{u}} ^n||^2\right] \le C. $$

Let $\tau$ be small enough, such that $1-C_2\tau>0, C_0\mu -C_2\beta \tau >0$, then we obtain from (30) that

31 $$ ||{\mathbf{u}} ^{n}|| \le C,\quad ||\delta _x{\mathbf{u}} ^n|| \le C.$$

An application of Lemma 4 yields

32 $$ ||{\mathbf{u}} ^n||_{\infty }\le C.$$

### *Remark 2*

Theorem 2 implies that scheme (11)–(14) is unconditionally stable.

### **Theorem 3**

*The difference scheme* (11) *is uniquely solvable*.

### *Proof*

By the mathematical induction. It is obvious that ${\mathbf{u}} ^0$ is uniquely determined by (13). We can choose a fourth-order method to compute ${\mathbf{u}} ^1$ [such as C–N scheme (12)]. Assuming that ${\mathbf{u}} ^1,\ldots ,{\mathbf{u}} ^n$ are uniquely solvable, consider ${\mathbf{u}} ^{n+1}$ in (11) which satisfies

33 $$ \frac{1}{2\tau }{} {\mathbf{u}} ^{n+1}-\frac{1}{2\tau }\mu {\varvec{G}}\delta _x\delta _{\bar{x}}{} {\mathbf{u}} ^{n+1} +\frac{1}{8}\alpha {\varvec{H}}\left[ \delta _{\hat{x}}{} {\mathbf{u}} ^{n+1}\left( {\mathbf{u}} ^{n}\right) ^2+\delta _{\hat{x}}\left( \left( {\mathbf{u}} ^n\right) ^2{\mathbf{u}} ^{n+1}\right) \right] =0. $$

Doing in (33) the inner product of with ${\mathbf{u}} ^{n+1}$ and using Lemma 1 yield

34 $$ \dfrac{1}{2\tau }||{\mathbf{u}} ^{n+1}||^2+\dfrac{1}{2\tau }\mu ||{\varvec{R}}\delta _x{\mathbf{u}} ^{n+1}||^2+\left( \phi \left( {\mathbf{u}} ^n,{\mathbf{u}} ^{n+1}\right) ,{\mathbf{u}} ^{n+1}\right) =0, $$

where $$\phi ({\mathbf{u}} ^n,{\mathbf{u}} ^{n+1})=\frac{1}{8}\alpha {\varvec{H}}[\delta _{\hat{x}}{} {\mathbf{u}} ^{n+1}({\mathbf{u}} ^{n})^2+\delta _{\hat{x}}(({\mathbf{u}} ^n)^2{\mathbf{u}} ^{n+1})]$$.

Similarly to the proof of (26), we obtain

35 $$ \left( \phi \left( {\mathbf{u}} ^n,{\mathbf{u}} ^{n+1}\right) ,{\mathbf{u}} ^{n+1}\right) =0.$$

This together with (34) gives that

36 $$ \dfrac{1}{2\tau }||{\mathbf{u}} ^{n+1}||^2+\dfrac{1}{2\tau }\mu ||{\varvec{R}}\delta _x{\mathbf{u}} ^{n+1}||^2=0.$$

This implies that there uniquely exists trivial solution satisfying Eq. (33). Hence, ${\mathbf{u}} ^{n+1}$ in (11) is uniquely solvable. This completes the proof of Theorem 3.

## Convergence and stability of the difference scheme

First, we shall consider the truncation error of the difference scheme (11)–(14). Let $v^n_j=u(x_j,t_n)$. We define the truncation error as follows:

37 $$\begin{aligned}{\varvec{r}}^n&=\delta _{\hat{t}}{} {\mathbf{v}} ^n-\mu {\varvec{G}}\delta _x\delta _{\bar{x}}\delta _{\hat{t}}{} {\mathbf{v}} ^n+{\varvec{H}}\delta _{\hat{x}}{} {\mathbf{v}} ^n +\dfrac{1}{4}\alpha {\varvec{H}}\left\{ \left(\delta_{\hat{x}}\bar{\mathbf{v}}^n\right) \left( {\mathbf{v}} ^n\right)^2+\delta _{\hat{x}}\left[ \left({\mathbf{v}} ^n\right) ^2\bar{\mathbf{v }}^n\right] \right\}\\ &\quad 1\le n\le N-1,\end{aligned}$$

38 $$\begin{aligned}\varvec{\sigma }^0&=\delta _t{\mathbf{v}} ^0-\mu {\varvec{G}}\delta _x\delta _{\bar{x}}\delta _t{\mathbf{v}} ^0+{\varvec{H}}\delta _{\hat{x}}{} {\mathbf{v}} ^{0+\frac{1}{2}} +\dfrac{1}{4}\alpha {\varvec{H}}\left\{ \left( \delta _{\hat{x}}{} {\mathbf{v}} ^{0+\frac{1}{2}}\right) \left( {\mathbf{v}} ^{0+\frac{1}{2}}\right)^2\right.\\ &\left.\quad +\delta _{\hat{x}}\left[ \left( {\mathbf{v}} ^{0+\frac{1}{2}}\right) ^3\right] \right\},\end{aligned} $$

39 $${\mathbf{v}} ^0={\mathbf{u}} _0,\quad 1\le j\le J-1, $$

40 $$v^n_0=v^n_J=0,\quad 0\le n\le N.$$

Using Taylor expansion and considering the construction of the difference scheme of (7)–(10), we know the accuracy of (7)–(10) is $O(\tau ^2+h^4)$. The scheme (11)–(14) is equivalent to (7)–(10). Then we have that $|{\varvec{r}}^n|=O(\tau ^2+h^4),|\varvec{\sigma }^0|=O(\tau ^2+h^4)$ hold if $\tau ,h\rightarrow 0$.

Next, we shall discuss the convergence and stability of the scheme (11)–(14).

### **Lemma 5**

(Discrete Gronwall inequality Zhou 1990) *Suppose that the discrete mesh function*$\{w^n|n=1,2,\ldots ,N; N\tau =T\}$*satisfies recurrence formula*

$$ w^n-w^{n-1}\le A\tau w^n+B\tau w^{n-1}+C_n\tau , $$

where *A*,*B* and $C_n$ ($n=1,\ldots ,N$) are nonnegative constants. Then

$$ ||w^n||_\infty \le \left( w^0+\tau \sum _{k=1}^{N}C_k\right) e^{2(A+B)T},$$

where $\tau$ is small, such that $(A+B)\tau \le \frac{N-1}{2N} (N > 1)$.

### **Theorem 4**

*Assume that*$u_0$*is sufficiently smooth and*$u(x,t)\in C^{5,3}_{x,t}$*, then the solution*${\mathbf{u}} ^n$*of the scheme* (10)–(12) *converges to the solution of the IBV problem* (3)–(5) *and the rate of convergence is*$O(\tau ^2+h^4)$*by the*$||\cdot ||_{\infty }$*norm*.

### *Proof*

Let ${\mathbf{e}} ^n={\mathbf{v}} ^n-{\mathbf{u}} ^n$. From (37) to (40) and (11) to (14), we have

41 $$\begin{aligned}{\varvec{r}}^n&=\delta _{\hat{t}}{} {\mathbf{e}} ^n-\mu {\varvec{G}}\delta _x\delta _{\bar{x}}\delta _{\hat{t}}{} {\mathbf{e}} ^n+{\varvec{H}}\delta _{\hat{x}}{} {\mathbf{e}} ^n +\dfrac{1}{4}\alpha {\varvec{H}}\left\{ \left( \delta _{\hat{x}}\bar{\mathbf{v }}^n\right) \left( {\mathbf{v}} ^n\right) ^2+\delta _{\hat{x}}\left[ \left( {\mathbf{v}} ^n\right) ^2\bar{\mathbf{v }}^n\right] \right\} \nonumber \\&\quad  -\dfrac{1}{4}\alpha {\varvec{H}}\left\{ \left( \delta _{\hat{x}}\bar{{\mathbf{u}}}^n\right) \left( {\mathbf{u}} ^n\right) ^2+\delta _{\hat{x}}\left[ \left( {\mathbf{u}} ^n\right) ^2\bar{{\mathbf{u}}}^n\right] \right\} ,\quad 1\le n\le N-1, \end{aligned}$$

42 $$\begin{aligned}\varvec{\sigma }^0&=\delta _t{\mathbf{e}} ^0-\mu {\varvec{G}}\delta _x\delta _{\bar{x}}\delta _t{\mathbf{e}} ^0+{\varvec{H}}\delta _{\hat{x}}{} {\mathbf{e}} ^{0+\frac{1}{2}} +\dfrac{1}{4}\alpha {\varvec{H}}\left\{ \left( \delta _{\hat{x}}{} {\mathbf{v}} ^{0+\frac{1}{2}}\right) \left( {\mathbf{v}} ^{0+\frac{1}{2}}\right) ^2+\delta _{\hat{x}}\left[ \left( {\mathbf{v}} ^{0+\frac{1}{2}}\right) ^3\right] \right\} \nonumber \\&\quad  -\dfrac{1}{4}\alpha {\varvec{H}}\left\{ \left( \delta _{\hat{x}}{} {\mathbf{u}} ^{0+\frac{1}{2}})({\mathbf{u}} ^{0+\frac{1}{2}}\right) ^2+\delta _{\hat{x}}\left[ \left( {\mathbf{u}} ^{0+\frac{1}{2}}\right) ^3\right] \right\} , \end{aligned}$$

43 $$\begin{aligned}&{\mathbf{e}} ^0=0,\quad 1\le j\le J-1, \end{aligned}$$

44 $$\begin{aligned}&e^n_0=e^n_J=0,\quad 0\le n\le N. \end{aligned}$$

Doing in (41) the inner product with $2\bar{\mathbf{e }}^{n}$ (i.e. ${\mathbf{e}} ^{n+1}+{\mathbf{e}} ^{n-1}$) and using Lemma 1, we obtain

45 $$\begin{aligned} \left( {\varvec{r}}^n,2\bar{\mathbf{e }}^{n}\right)&=\frac{1}{2\tau }\left( ||{\mathbf{e}} ^{n+1}||^2-||{\mathbf{e}} ^{n-1}||^2\right) +\frac{1}{2\tau }\mu \left( ||{\varvec{R}}\delta _x{\mathbf{e}} ^{n+1}||^2-||{\varvec{R}}\delta _x{\mathbf{e}} ^{n-1}||^2\right) \nonumber \\&\quad + h\sum _{j=1}^{J-1}\left( {\varvec{S}}\delta _{\hat{x}}e_j^{n}\right) {\varvec{S}}\left( e^{n+1}_j+e_j^{n-1}\right) +\left( P+Q,2\bar{\mathbf{e }}^{n}\right) , \end{aligned}$$

where $$P=\frac{1}{4}\alpha {\varvec{H}}[(\delta _{\hat{x}}\bar{\mathbf{v }}^n)({\mathbf{v}} ^n)^2-(\delta _{\hat{x}}\bar{{\mathbf{u}}}^n)({\mathbf{u}} ^n)^2]$$, $$Q=\frac{1}{4}\alpha {\varvec{H}}\{[\delta _{\hat{x}}[({\mathbf{v}} ^n)^2\bar{\mathbf{v }}^n]-\delta _{\hat{x}}[({\mathbf{u}} ^n)^2\bar{{\mathbf{u}}}^n]\}$$.

Computing the fourth term of right-hand side of (45) and using Theorem 2, Lemma 3 yield

46 $$\begin{aligned} \left( P,2\bar{\mathbf{e }}^{n}\right)&=\frac{1}{2}\alpha \left( {\varvec{H}}\left[ \left( \delta _{\hat{x}}\bar{\mathbf{v }}^n\right) \left( {\mathbf{v}} ^n\right) ^{2}-\left( \delta _{\hat{x}}\bar{{\mathbf{u}}}^n\right) \left( {\mathbf{u}} ^n\right) ^{2}\right] ,\bar{\mathbf{e }}^{n}\right) \nonumber \\&=\frac{1}{2}\alpha \left( {\varvec{H}}\left[ \left( \delta _{\hat{x}}\bar{\mathbf{e }}^n\right) \left( {\mathbf{v}} ^n\right) ^{2}+\left( \delta _{\hat{x}}\bar{{\mathbf{u}}}^n\right) \left( \left( {\mathbf{v}} ^n\right) ^{2}-\left( {\mathbf{u}} ^n\right) ^{2}\right) \right] ,\bar{\mathbf{e }}^{n}\right) \nonumber \\&=\frac{1}{2}\alpha h\left\{ \sum _{j=1}^{J-1}{\varvec{S}}\left( \delta _{\hat{x}}\bar{e}^{n}_j\right) \left( v^{n}_j\right) ^{2}{\varvec{S}}\bar{e}^{n}_j +\sum _{j=1}^{J-1}{\varvec{S}}\left( \delta _{\hat{x}}\bar{u}^{n}_j\right) \left[ \left( v^{n}_j\right) ^{2}-\left( u^{n}_j\right) ^{2}\right] {\varvec{S}}\bar{e}^{n}_j\right\} \nonumber \\&=\frac{1}{2}\alpha h\sum _{j=1}^{J-1}{\varvec{S}}\left( \delta _{\hat{x}}\bar{e}^{n}_j\right) \left( v^{n}_j\right) ^{2}{\varvec{S}}\bar{e}^{n}_j +\frac{1}{2}\alpha h\sum _{j=1}^{J-1}{\varvec{S}}\left( \delta _{\hat{x}}\bar{u}^{n}_j\right) \left[ e_j^{n}\left( v^{n}_j-u^{n}_j\right) \right] {\varvec{S}}\bar{e}^{n}_j\nonumber \\&\le C\left( ||{\varvec{S}}\delta _{\hat{x}}\bar{\mathbf{e }}^{n}||^2+||{\varvec{S}}{} {\mathbf{e}} ^{n}||^2+||{\varvec{S}}\bar{\mathbf{e }}^{n}||^2\right) \nonumber \\&\le C\left( ||{\varvec{R}}\delta _x{\mathbf{e}} ^{n+1}||^2+||{\varvec{R}}\delta _x{\mathbf{e}} ^{n-1}||^2+||{\mathbf{e}} ^{n+1}||^2+||{\mathbf{e}} ^{n}||^2+||{\mathbf{e}} ^{n-1}||^2\right) . \end{aligned}$$

Similarly, we have that

47 $$ \left( Q,2\bar{e}^{n}\right) \le C\left( ||{\varvec{R}}\delta _x{\mathbf{e}} ^{n+1}||^2+||{\varvec{R}}\delta _x{\mathbf{e}} ^{n-1}||^2+||{\mathbf{e}} ^{n+1}||^2+||{\mathbf{e}} ^{n}||^2+||{\mathbf{e}} ^{n-1}||^2\right) . $$

In addition, it is obvious that

48 $$ \left( {\varvec{r}}^n,2\bar{\mathbf{e }}^{n}\right) \le ||{\varvec{r}}^n||^2 +\frac{1}{2}\left( ||{\mathbf{e}} ^{n+1}||^2+||{\mathbf{e}} ^{n-1}||^2\right) , $$

49 $$\begin{aligned} h\sum _{j=1}^{J-1}\left( {\varvec{S}}\delta _{\hat{x}}e_j^{n}\right) {\varvec{S}}\left( e^{n+1}_j+e_j^{n-1}\right)&\le ||{\varvec{S}}\delta _x{\mathbf{e}} ^n||^2+\frac{1}{2}\left( ||{\mathbf{e}} ^{n+1}||^2+||{\mathbf{e}} ^{n-1}||^2\right) \nonumber \\&\le C||{\varvec{R}}\delta _x{\mathbf{e}} ^n||^2+\frac{1}{2}\left( ||{\mathbf{e}} ^{n+1}||^2+||{\mathbf{e}} ^{n-1}||^2\right) . \end{aligned}$$

It follows from (45) to (49) that

50 $$\begin{aligned}&\frac{1}{2\tau }\left( ||{\mathbf{e}} ^{n+1}||^2-||{\mathbf{e}} ^{n-1}||^2\right) +\frac{1}{2\tau }\left( ||{\varvec{R}}\delta _x{\mathbf{e}} ^{n+1}||^2-||{\varvec{R}}\delta _x{\mathbf{e}} ^{n-1}||^2\right) \nonumber \\&\quad \le ||{\varvec{r}}^n||^2+C\left[ ||{\varvec{R}}\delta _x{\mathbf{e}} ^{n+1}||^2+||{\varvec{R}}\delta _x{\mathbf{e}} ^n||^2+||{\varvec{R}}\delta _x{\mathbf{e}} ^{n-1}||^2+||{\mathbf{e}} ^{n+1}||^2+||{\mathbf{e}} ^{n}||^2+||{\mathbf{e}} ^{n-1}||^2\right] . \end{aligned}$$

Let $B^n=\frac{1}{2}(||{\mathbf{e}} ^{n+1}||^2+||{\mathbf{e}} ^{n}||^2)+\frac{1}{2}(||{\varvec{R}}\delta _x{\mathbf{e}} ^{n+1}||^2+||{\varvec{R}}\delta _x{\mathbf{e}} ^{n}||^2)$, then (50) can be written as follows:

51 $$ B^{n}-B^{n-1}\le \tau ||{\varvec{r}}^n||^2+C\tau \left( B^{n}+B^{n-1}\right) .$$

By Lemma 5, it can immediately be obtained that

52 $$ B^n\le \left( B^0+T\sup _{l\le n\le N}||{\varvec{r}}^n||^2\right) e^{CT}. $$

Taking the inner product in (42) with ${\mathbf{e}} ^1$, we have

53 $$ \left( \varvec{\sigma }^0,{\mathbf{e}} ^1\right) =\frac{1}{\tau }||{\mathbf{e}} ^1||^2+\frac{1}{\tau }\mu ||{\varvec{R}}\delta _x{\mathbf{e}} ^1||^2+\frac{1}{2}\left( {\varvec{H}}\delta _{\hat{x}}{} {\mathbf{e}} ^1,{\mathbf{e}} ^1\right) +\left( I,{\mathbf{e}} ^1\right) +\left( II,{\mathbf{e}} ^1\right) , $$

where $$I=\frac{1}{4}\alpha {\varvec{H}}[(\delta _{\hat{x}}{} {\mathbf{v}} ^{0+\frac{1}{2}})({\mathbf{v}} ^{0+\frac{1}{2}})^2-(\delta _{\hat{x}}{} {\mathbf{u}} ^{0+\frac{1}{2}})({\mathbf{u}} ^{0+\frac{1}{2}})^2]$$, $$II=\frac{1}{4}\alpha {\varvec{H}}\{\delta _{\hat{x}}[({\mathbf{v}} ^{0+\frac{1}{2}})^3]-\delta _{\hat{x}}[({\mathbf{u}} ^{0+\frac{1}{2}})^3]\}$$.

Notice that

54 $$ \frac{1}{2}\left( {\varvec{H}}\delta _{\hat{x}}{} {\mathbf{e}} ^1,{\mathbf{e}} ^1\right) =0. $$

From the discrete initial condition (43), we know that ${\mathbf{e}} ^0=0$. Computing the fourth term of right-hand side in (53) and using Theorem 2, Lemma 3 yield

55 $$\begin{aligned} \left( I,{\mathbf{e}} ^1\right)&=\left( \frac{1}{4}\alpha {\varvec{H}}\left[ \left( \delta _{\hat{x}}{} {\mathbf{v}} ^{0+\frac{1}{2}}\right) \left( {\mathbf{v}} ^{0+\frac{1}{2}}\right) ^2- \left( \delta _{\hat{x}}{} {\mathbf{u}} ^{0+\frac{1}{2}}\right) \left( {\mathbf{u}} ^{0+\frac{1}{2}}\right) ^2\right] ,{\mathbf{e}} ^1\right) \nonumber \\&=\frac{1}{8}\alpha \left( {\varvec{S}}\left[ \left( \delta _{\hat{x}}{} {\mathbf{e}} ^1\right) \left( {\mathbf{v}} ^{0+\frac{1}{2}}\right) ^2+ \left( \delta _{\hat{x}}{} {\mathbf{u}} ^{0+\frac{1}{2}}\right) {\mathbf{e}} ^1 \left( {\mathbf{v}} ^{0+\frac{1}{2}}+{\mathbf{u}} ^{0+\frac{1}{2}}\right) \right] ,{\varvec{S}}{} {\mathbf{e}} ^1\right) \nonumber \\&\le C\left( ||{\varvec{S}}\delta _x{\mathbf{e}} ^1||^2+||{\varvec{S}}{} {\mathbf{e}} ^1||^2\right) \nonumber \\&\le C\left( ||\delta _x{\mathbf{e}} ^1||^2+||{\mathbf{e}} ^1||\right) . \end{aligned}$$

Similarly, we get

56 $$ \left( II,{\mathbf{e}} ^1\right) \le C\left( ||\delta _x{\mathbf{e}} ^1||^2+||{\mathbf{e}} ^1||\right) . $$

It follows from (53) to (56) that

57 $$ \frac{1}{\tau }||{\mathbf{e}} ^1||^2+\frac{1}{\tau }\mu ||{\varvec{R}}\delta _x{\mathbf{e}} ^1||^2\le \left( \varvec{\sigma }^0,{\mathbf{e}} ^1\right) +C\left( ||\delta _x{\mathbf{e}} ^1||^2+||{\mathbf{e}} ^1||\right) . $$

This together with $(\varvec{\sigma }^0,{\mathbf{e}} ^1)\le \frac{1}{2}(||\varvec{\sigma }^0||^2+||{\mathbf{e}} ^1||^2)$ and Lemma 3 gives that

58 $$ \left( \frac{1}{\tau }-\frac{1}{2}-C\right) ||{\mathbf{e}} ^1||^2+ \left( \frac{1}{\tau }C_0\mu -C\right) ||\delta _x{\mathbf{e}} ^1||^2\le \frac{1}{2}||\varvec{\sigma }^0||^2.$$

Let $\tau$ be small enough, such that $\frac{1}{\tau }-\frac{1}{2}-C> 0, \frac{1}{\tau }C_0\mu -C> 0$. Then we obtain from (58) that

59 $$ ||{\mathbf{e}} ^1||^2\le K_1 [O(\tau ^2+h^4)]^2,\quad ||\delta _x{\mathbf{e}} ^1||^2\le K_2 \left[ O\left( \tau ^2+h^4\right) \right] ^2, $$

where $$K_1=\frac{1}{\frac{2}{\tau }-1-2C}, K_2=\frac{1}{\frac{2}{\tau }C_0\mu -2C}$$.

It follows from Lemma 3 that

60 $$ ||{\varvec{R}}\delta _x{\mathbf{e}} ^1||^2\le C_1K_2 \left[ O\left( \tau ^2+h^4\right) \right] ^2. $$

This implies that

61 $$ B^0=\left[ O\left( \tau ^2+h^4\right) \right] ^2. $$

It follows from (52) that

62 $$ B^n\le C\left[ O\left( \tau ^2+h^4\right) \right] ^2, $$

which together with Lemmas 3 and 4, and the definition of $B^n$ gives that

63 $$ ||{\mathbf{e}} ^n||_{\infty }\le C\cdot O\left( \tau ^2+h^4\right) . $$

This completes the proof of Theorem 4.

Similarly, we can prove stability of the difference solution.

### **Theorem 5**

*Under the conditions of* Theorem 4*, the solution*${\mathbf{u}} ^n$*of the scheme* (11)–(14) *is unconditionally stable by the*$||\cdot ||_{\infty }$*norm*.

## Numerical experiments

In this section, we conduct some numerical experiments to verify our theoretical results obtained in the previous sections. In order to test whether the present scheme (11)–(14) exhibits the expect convergence rates in time and in space, we will measure the accuracy of the proposed scheme using the square norm errors and the maximum norm errors defined by

$$ e^n_{\epsilon _1}=||{\mathbf{v}} ^n-{\mathbf{u}} ^n||_{\infty },\quad e^n_{\epsilon _2}=||{\mathbf{v}} ^n-{\mathbf{u}} ^n||.$$

The exact solution of the IBV problem (3)–(5) has the following form (Gardner et al. 1997):

64 $$ u(x,t)=\sqrt{\frac{6c}{\alpha }}\hbox {sech} \left[ \sqrt{\frac{c}{\mu (c+1)}}(x-(c+1)t-x_0)\right] ,$$

where $x_0, c$ are arbitrary constants.

It follows from (64) that the initial-boundary value problem (3)–(5) is consistent to the initial value problem (3) and (4) for $-x_l\gg 0$, $x_r\gg 0$. In the following numerical experiments, we take $x_l$ = −40, $x_r=60$, $T=10.$

The IBV problem (3)–(5) has another two invariants (Gardner et al. 1997):

65 $$ Q(t)=\int _{x_l}^{x_r} u(x,t) {\mathrm{d}}x\simeq h\sum _{j=1}^{J-1}u_j^n, $$

66 $$ \tilde{E}(t)=\int _{x_l}^{x_r} \left[ u^4-\frac{6\mu }{\alpha }u^2_x\right] \,{\mathrm{d}}x\simeq h\sum _{j=1}^{J-1}\left[ \left( u_j^n\right) ^4-\frac{6\mu }{\alpha }\left( (u_x)_j^n\right) ^2\right] . $$

The initial condition of the studied model is obtained from (64) with the parameters $x_0$, *c*, $\alpha$ and *μ*:

67 $$ u(x,0)=\sqrt{\frac{6c}{\alpha }}\hbox {sech} \left[ \sqrt{\frac{c}{\mu (c+1)}}(x-x_0)\right] .$$

**Fig. 1:**
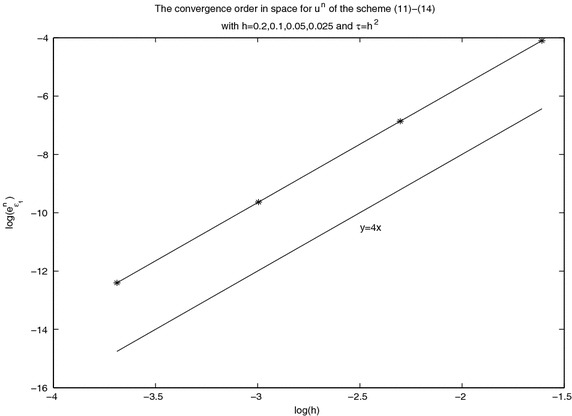
Spatial convergence order in maximal norm for $u^n$ at $t=10$ with different *h* and $\tau$ computed by the scheme (11)–(14)

**Fig. 2:**
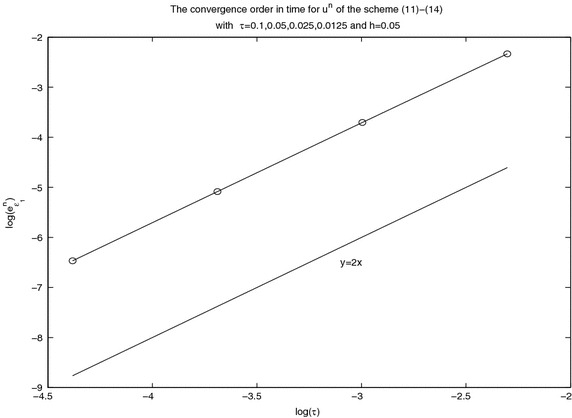
Temporal convergence order in maximal norm for $u^n$ at $t=10$ with different *h* and $\tau$ computed by the scheme (11)–(14)

In computations, we always choose the parameter $x_0=0$. Take the parameters $c=\alpha =\mu =1$. To verify the accuracy $O(\tau ^2+h^4)$ in the spatial direction, we take $\tau =h^2$. And we choose *h* small enough to verify the second-order accuracy in the temporal direction. The convergence order figure of $\log (e^n)$–$\log (h)$ with $\tau =h^2$ and the one of $\log (e^n)$–$\log (\tau )$ with *h* small enough are given in Figs. 1 and 2 under various mesh steps *h* and $\tau$ at $t=10$. From Figs. 1 and 2, it is obvious that the scheme (11)–(14) is convergent in maximum norm, and the convergence order is $O(\tau ^2+h^4).$

The errors in the sense of $L_\infty$-norm and $L_2$-norm of the numerical solutions ${\mathbf{u}} ^n$ of the scheme (11) are listed on Tables 1 and 2. Tables 1 and 2 shows good stability of the numerical solutions and also verify the scheme in present paper is efficient and of high accuracy.

We have shown in Theorem 1 that the numerical solution ${\mathbf{u}} ^n$ of the scheme (11) satisfies the conservative property (23). The values of $E^n$, *Q*$\tilde{E}$ for the scheme (11) are presented in Table 3 under steps $h=0.1$ and $\tau =0.01$. It is easy to see from Table 3 that the scheme (11) preserves the discrete conservative properties very well, thus it can be used to computing for a long time.

**Table 1:** The errors for numerical solutions $u^n$ of the scheme (11)–(14) at different time *t* with various *h* and $\tau =h^2$

*t*	$h=0.2$		0.1		0.05		0.025	
	$e^n_{\epsilon _1}$	$e^n_{\epsilon _2}$	$e^n_{\epsilon _1}$	$e^n_{\epsilon _2}$	$e^n_{\epsilon _1}$	$e^n_{\epsilon _2}$	$e^n_{\epsilon _1}$	$e^n_{\epsilon _2}$
2	3.7504e−3	6.8705e−3	2.3827e−4	4.3361e−4	1.4930e−5	2.7153e−5	9.3409e−7	1.6997e−6
4	7.1325e−3	1.3403e−2	4.5011e−4	8.438e−4	2.8167e−5	5.2812e−5	1.7639e−6	3.3089e−6
6	1.0303e−2	1.9666e−2	6.5000e−4	1.2367e−3	4.0665e−5	7.7372e−5	2.5487e−6	4.8528e−6
8	1.3432e−2	2.5820e−2	8.4767e−4	1.6227e−3	5.3023e−5	1.0151e−4	3.3268e−6	6.3733e−6
10	1.6551e−2	3.1935e−2	1.0447e−3	2.0063e−3	6.5345e−5	1.2549e−4	4.1045e−6	7.8878e−6

**Table 2:** The errors for numerical solutions $u^n$ of the scheme (11)–(14) at different time *t* with various $\tau$ when $h=0.05$

*t*	$\tau =0.1$		0.05		0.025		0.0125	
	$e^n_{\epsilon _1}$	$e^n_{\epsilon _2}$	$e^n_{\epsilon _1}$	$e^n_{\epsilon _2}$	$e^n_{\epsilon _1}$	$e^n_{\epsilon _2}$	$e^n_{\epsilon _1}$	$e^n_{\epsilon _2}$
2	2.1201e−2	3.9697e−2	5.4220e−3	1.0131e−2	1.3673e−3	2.5533e−3	3.4332e−4	6.4146e−4
4	4.1031e−2	7.8091e−2	1.0443e−2	1.9881e−2	2.6281e−3	5.0027e−3	6.5986e−4	1.2555e−3
6	5.9886e−2	1.1514e−1	1.5225e−2	2.9270e−2	3.8276e−3	7.3585e−3	9.6034e−4	1.8457e−3
8	7.8493e−2	1.5154e−1	1.9948e−2	3.8497e−2	5.0129e−3	9.6738e−3	1.2572e−3	2.4257e−3
10	9.7041e−2	1.8768e−1	2.4657e−2	4.7665e−2	6.1945e−3	1.1974e−2	1.5533e−3	3.0020e−3

**Table 3:** Discrete conservative properties of the scheme (11)–(14) when $h=0.1$ and $\tau =0.01$

*t*	$E^n$	*Q*	$\tilde{E}$
2	19.7989864026379	10.8827962014572	50.9215819473395
4	19.7989864025761	10.8827962035498	50.9215813909925
6	19.7989864025143	10.8827962044781	50.9215812185741
8	19.7989864024516	10.8827962047988	50.9215811567766
10	19.7989864023892	10.8827962049056	50.9215811324460

**Fig. 3:**
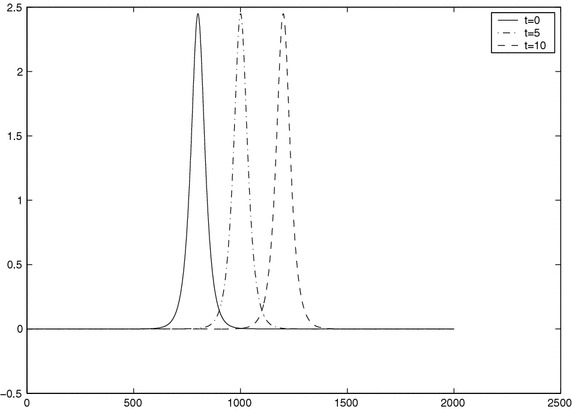
Exact solutions *u*(*x*, *t*) at $t=0$ and numerical solutions $u^n$ computed by the scheme (11)–(14) with $h=0.05, \tau =0.0025$ at $t=5$ and 10

The curves of the solitary wave with time computed by scheme (11) with $h=0.05$ and $\tau =0.0025$ are given in Fig. 3; the waves at $t=5$ and 10 agree with the ones at $t=0$ quite well, which also demonstrate the accuracy and efficiency of the scheme in present paper.

To compare the numerical results with other results shown in previous studies, we denote the proposed scheme in Akbari and Mokhtari (2014) as Scheme I with $p=2, \mu =\varepsilon =1$ and $d=\frac{1}{3}$. Denote the present scheme (11) with $c=\frac{1}{3}, \alpha =\mu =1$ as Scheme II. The corresponding errors in the sense of $L_\infty$-norm and CPU time are listed on Table 4 under different mesh steps *h* and $\tau$. From Table 4, we get that a fourth-order three-level linear scheme as accurate as Scheme I which is a two-level one.

**Table 4:** The maximal errors for numerical solutions $u^n$ of Scheme I and II at time $t=10$ with various *h* and $\tau$

$(h,\tau )$	(0.1, 0.2)	(0.05, 0.05)	(0.025, 0.0125)	(0.0125, 0.003125)
Scheme I	3.11496e−2	1.53986e−3	8.84019e−5	5.40468e−6
CPU time (s)	0.929105	2.412442	19.031491	255.135799
Scheme II	2.91603e−2	1.87511e−3	1.17545e−4	7.35116e−6
CPU time (s)	0.727532	2.229997	17.104052	239.567755

## Conclusion

In this paper, an attempt has been made to construct a new numerical scheme to solve the initial-boundary problem of the MRLW equation, which has the following advantages: Coupling with the matrix theory, we convert the proposed scheme into the vector difference one. The new scheme is high-accuracy which has the accuracy of $O(\tau ^2+h^4)$; The new scheme is conservative and preserves the original conservative properties; The coefficient matrices of the scheme is symmetric and tridiagonal, so Thomas algorithm can be employed to solve it effectively. Some numerical results are reported to show the efficiency and accuracy of the scheme.
